# Temperature-dependent breakdown of hydrogen peroxide-treated ZnO and TiO_2_ nanoparticle agglomerates

**DOI:** 10.3762/bjnano.6.193

**Published:** 2015-09-14

**Authors:** Sinan Sabuncu, Mustafa Çulha

**Affiliations:** 1 Genetics and Bioengineering Department, Faculty of Engineering, Yeditepe University, Istanbul 34755, Turkey

**Keywords:** agglomeration, hydrogen peroxide, metal oxide nanoparticles, TiO_2_, ZnO

## Abstract

Metal oxide nanoparticles (MONPs) are used in a variety of applications including drug formulations, paint, sensors and biomedical devices due to their unique physicochemical properties. One of the major problems with their widespread implementation is their uncontrolled agglomeration. One approach to reduce agglomeration is to alter their surface chemistry with a proper functionality in an environmentally friendly way. In this study, the influence of hydrogen peroxide (H_2_O_2_) treatment on the dispersion of ZnO and TiO_2_ nanoparticle (NP) agglomerates as a function of temperature is studied. The H_2_O_2_ treatment of the MONPs increases the density of hydroxyl (–OH) groups on the NP surface, as verified with FTIR spectroscopy. The influence of heating on the dispersion of H_2_O_2_-treated ZnO and TiO_2_ NPs is investigated using dynamic light scattering. The untreated and H_2_O_2_-treated ZnO and TiO_2_ NP suspensions were heated from 30 °C to 90 °C at 5 °C intervals to monitor the breakdown of large aggregates into smaller aggregates and individual nanoparticles. It was shown that the combined effect of hydroxylation and heating enhances the dispersion of ZnO and TiO_2_ NPs in water.

## Introduction

Dispersion of metal oxide nanoparticles (MONPs) in aqueous media has attracted a considerable amount of interest due to their potential application in drug systems [1], gene therapy [2], sensing [3], and paint and pigments [4]. Similar to other nanometer scale materials, they tend to agglomerate and form large aggregates during or after their preparation. The degree of the agglomeration is mostly governed by the synthesis method, which defines their surface properties. During the synthesis processes or in subsequent process steps, the agglomeration of primary particles occurs as a result of the weak bonding between NPs. These primary aggregates then form larger, strongly bonded micrometer size aggregates.

For their effective use, the NPs should remain stable and agglomeration should be avoided during any application process. This agglomeration can be chemically and physically prevented to a certain degree [5–6]. The use of surfactants is one of the most common ways of increasing the NP dispersion in aqueous media. Polymers such as polystyrene, poly(methyl methacrylate) (PMMA), and poly(acrylic acid) are also widely used to obtain dispersed NPs in aqueous environments [7–9]. Although the use of surfactants can provide better dispersion, they contaminate the NP suspension, which may limit the applications of the NPs.

Their dispersion in aqueous media can also be physically achieved after long ultrasonication processes (up to 60 h [10]). However, the long sonication time may also cause erosion or dissolution and the formation of cavities on the surface of the NPs [11–15]. Another external factor, temperature, can be utilized for this purpose. In this respect, in several studies, the temperature-dependent viscosity of nanofluids (which could be defined as solid–liquid materials established by the NP dispersions in the range of 1–100 nm) was examined [16]. The thermal conductivity and surface potential of the nanofluids were also studied [17–19].

The toxicity of NPs is another concern that is strongly related to their size, shape, and surface chemistry. Since the synthesis of NPs of a certain size and shape in large quantities is nearly impossible using current approaches, the surface chemistry can only be considered as an alternative to reduce the possible toxic effects. An appropriate functional group on the NP surface may improve biocompatibility and stability in various environments. In our previous study, we demonstrated that the treatment of ZnO NPs with hydrogen peroxide (H_2_O_2_) affected the surface properties and as a result the cytotoxicity of the ZnO NPs was found to decrease [20].

H_2_O_2_ is a powerful oxidizer and it is therefore routinely used in many cleaners and bleaches. Living systems can produce hydrogen peroxide as a product of oxidative metabolism processes. It is considered to be a dangerous product to living systems due to its possible damage to genetic material. In chemistry, it is used as an oxidizer in several reactions, such as decomposition, redox reactions, alkalinity and formation of peroxide compounds. However, the products formed from its decomposition (water and oxygen) are harmless. The treatment of MONPs with hydrogen peroxide is a new approach and there are only a few studies that focus on the reactivity of hydrogen peroxide with respect to the surface chemistry of MONPs [20–22]. These studies indicate that hydrogen peroxide treatment not only influences the surface properties of NPs but also changes the band gap of ZnO and TiO_2_ NPs and shifts the emission wavelength to blue wavelengths. Another study showed that the hydrogen peroxide treatment of TiO_2_ NPs increases the photocatalytic activity of the NPs [23].

The objective of this study is to disintegrate MONP agglomerates using heat and H_2_O_2_ treatment without the use of any additional chemicals. ZnO and TiO_2_ NPs were chosen due to their widespread use in several industrial applications. The H_2_O_2_ treatment not only eliminates any possible organic impurities remaining from the synthesis process but also increases the –OH group density on the NP surface. Therefore, the NPs exhibit better dispersion in aqueous media as evidenced from the change in their hydrodynamic radius.

## Experimental

### Chemicals

The ZnO (98 nm) and TiO_2_ (18 nm) NPs in powder form were a gift from Tec Star nanofiller consultants and providers. The hydrogen peroxide solution (34.5–36.5%) was purchased from Sigma Aldrich (Missouri, USA).

### TEM measurements

TEM images were obtained at the Marmara Research Institute (MAM) using a JEOL-2100 HR-TEM operating at 200 kV (LaB6 filament).

### Hydroxylation of ZnO and TiO_2_ NPs with H_2_O_2_ treatment

0.2 g of ZnO or TiO_2_ NPs were dispersed in 25 mL of H_2_O_2_ solution. The resulting NP suspension was dried on a preheated hot plate (Heidolph MR 3004, Germany), which was previously set to 300 hot plate scale and kept boiling until all of the solvent had evaporated. For the washing step, the resulting NP powder was dispersed in 10 mL of distilled water (dH_2_O) and dried on a hot plate, which was previously set to the 300 scale. Finally, the H_2_O_2_-treated NPs were obtained in powder form. The NPs were redispersed in dH_2_O and stored at room temperature.

### Fourier transform infrared spectroscopy (FTIR) measurements

FTIR analysis was performed using a Thermo Scientific, Nicolet iS50 FTIR instrument (Massachusetts, USA) in attenuated total reflectance mode with a diamond plate and ZnSe lens. The samples were dried at 90 °C on a hot plate before analysis.

### DLS analysis of the hydroxylated ZnO and TiO_2_ NPs

The hydrodynamic radius and zeta potential of the NPs in suspension were monitored using a Zetasizer NanoZS (Malvern, UK) instrument equipped with a 4 mW HeNe laser (633 nm). The hydroxylated ZnO or TiO_2_ NPs were added to dH_2_O (1 mg/mL). The prepared NP suspensions were sonicated for 30 min. Next, the sonicated suspensions were left to rest in order to obtain a saturated aqueous suspension. After the resting phase, the supernatant of the suspension was transferred to a tube and stored at room temperature. All experiments were performed from the same batch of NPs.

The agglomeration state of the untreated and hydroxylated NPs was evaluated over a temperature gradient from 30 to 90 °C with 5 °C intervals. Between each step, an interval of 5 min of equilibration time was used regardless of the instrument time to set the temperature. The suspension was placed into a folded capillary cell for zeta potential measurement and the zeta potential of the NPs was measured at 30 °C. All experiments were performed at least three times.

## Results and Discussion

**Figure 1:**
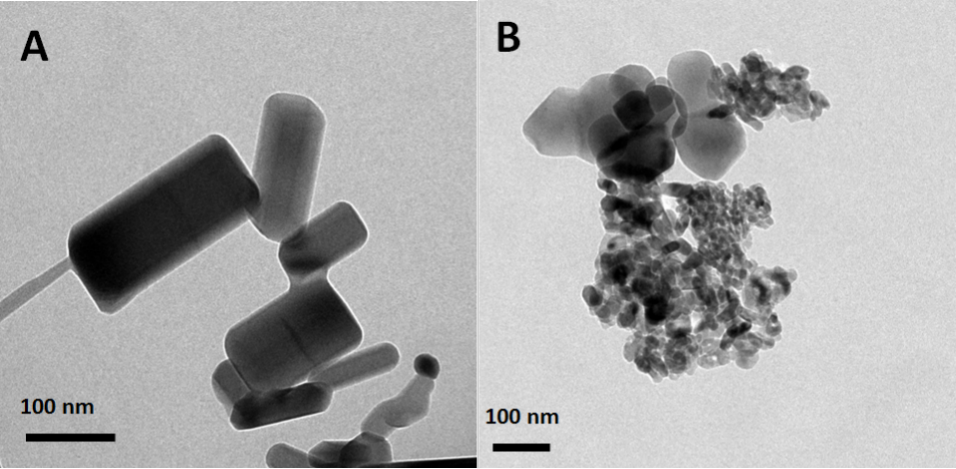
TEM images of pristine ZnO (a) and TiO_2_ (b) NPs.

Representative TEM images of the ZnO and TiO_2_ NPs are provided in Figure 1. As can be seen, both types of NPs are composed of NPs with a range of sizes. The average size of the ZnO and TiO_2_ NPs were 98 and 18 nm, respectively.

### Change in zeta potential after NP hydroxylation

**Figure 2:**
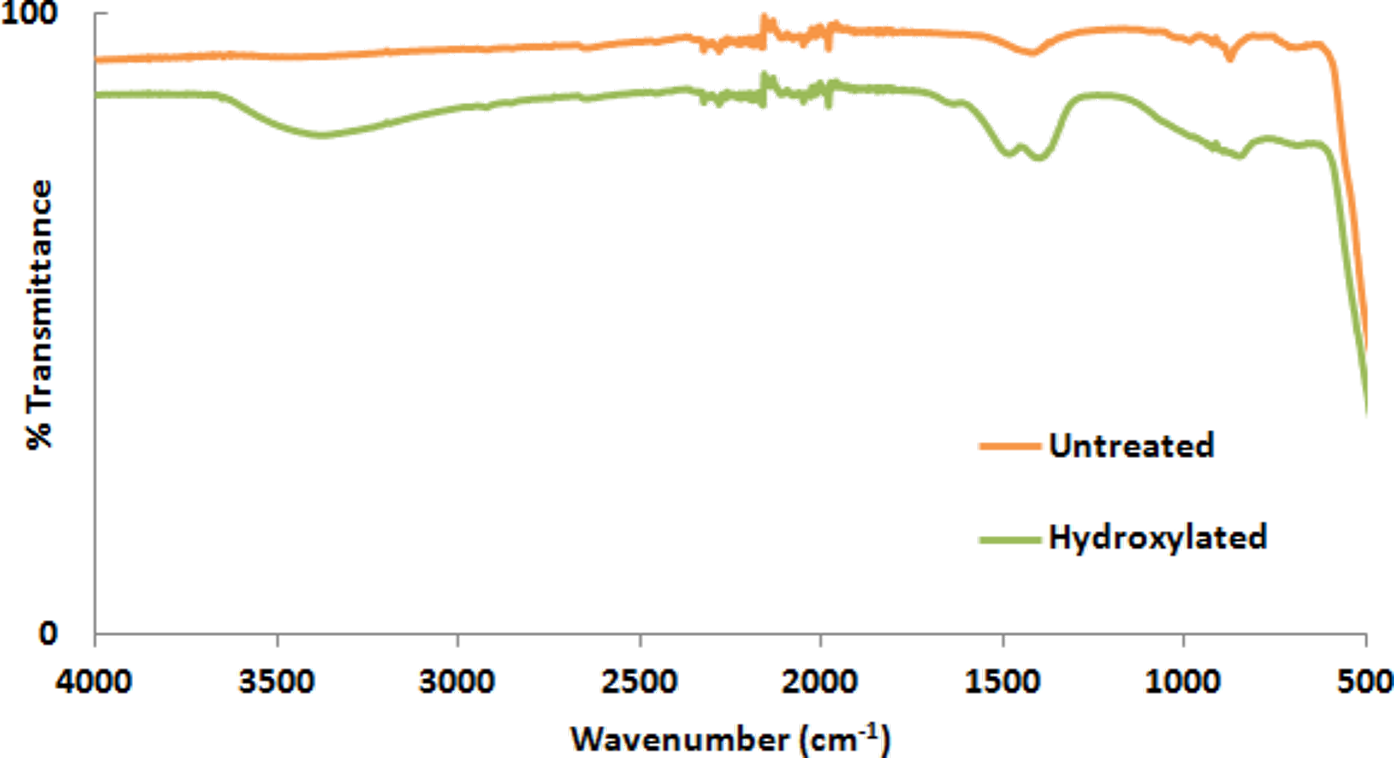
FTIR spectra of untreated and hydroxylated ZnO NPs.

**Figure 3:**
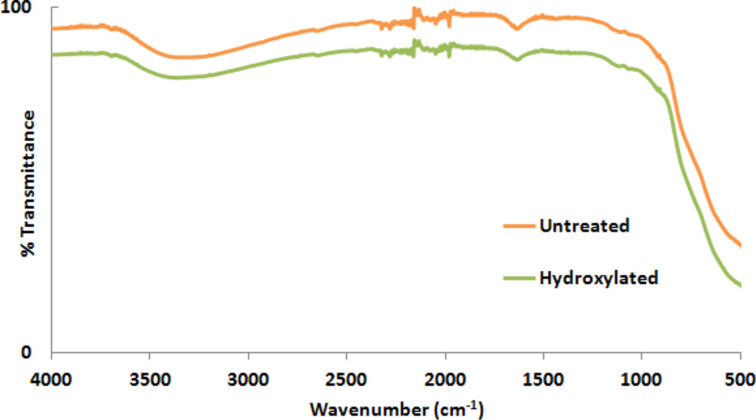
FTIR spectra of untreated and hydroxylated TiO_2_ NPs.

The zeta potential (ζ) of the NPs provides information about the surface charge of the particle in a solvent. The surface charge of a particle can vary with respect to the change in environment or any change in the surface chemistry. The proper treatment process of metal oxide NPs with H_2_O_2_ can reduce the activation energy of H_2_O_2_ resulting in the decomposition into H_2_O and O_2_, causing the release of OH^−^ or HO_2_ radicals. These radicals can remove impurities such as carbon and carbonate present on the surface and introduce hydroxyl groups (–OH) onto the surface [20,22,24]. Figure 2 and Figure 3 show the FTIR spectra of ZnO and TiO_2_ NPs before and after the H_2_O_2_ process, respectively. Figure 2 suggest that the intensity increase in the range of 3200–3600 cm^−1^ (the range of the characteristic IR absorption frequency of –OH) is due to the increased –OH density on the ZnO NP surface after H_2_O_2_ treatment. As can be seen in Figure 3, there is no significant difference in the intensity of the band attributed to the –OH groups in the range from 3200–3600 cm^−1^ since TiO_2_ NPs already have –OH groups on their surface. Although there is almost no difference in the FTIR spectra of TiO_2_ NPs before and after the hydrogen peroxide treatment, the change in zeta potential suggests a change in the surface properties of the TiO_2_ NPs. This might be due to the removal of low concentration organic impurities remaining on the NP surface from the synthesis. Table 1 shows the change in zeta potential of ZnO and TiO_2_ NPs before and after the hydroxylation process. The resulting hydroxylated NPs become rich in –OH groups on the NP surface, which causes in a shift of the zeta potential to more negative values relative to that of the untreated NPs.

**Table 1:** Zeta potential results of untreated ZnO NPs, TiO_2_ NPs, hydroxylated ZnO NPs, and hydroxylated TiO_2_ NPs.

	Zeta potential / mV (30 °C)

Untreated ZnO NPs	63.3 ± 0.70
Hydroxylated ZnO NPs	23.3 ± 1.13
Untreated TiO_2_ NPs	81.5 ± 0.212
Hydroxylated TiO_2_ NPs	32.5 ± 0.012

**Figure 4:**
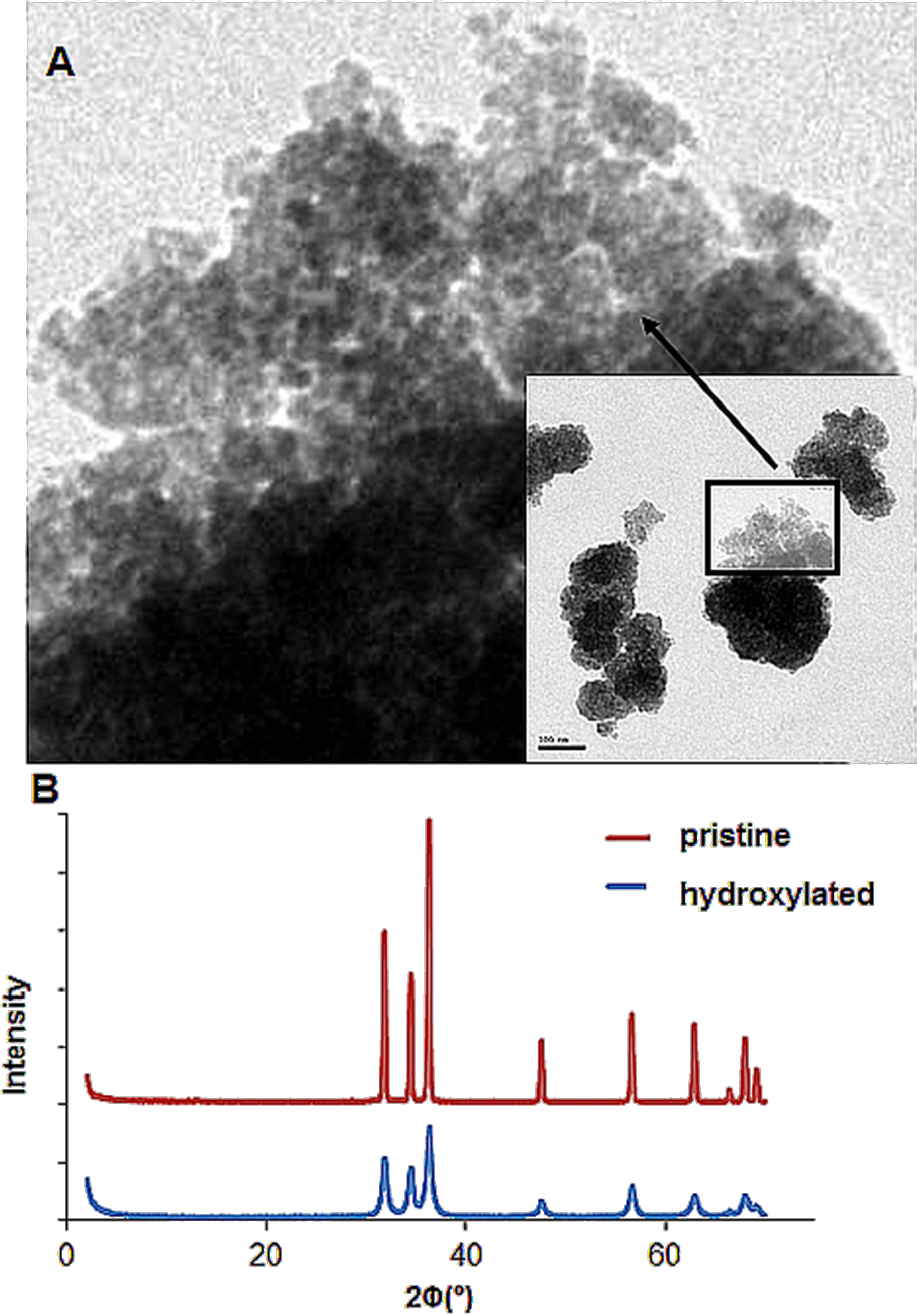
TEM images of H_2_O_2_-treated ZnO NPs (a) and XRD spectra before and after H_2_O_2_ treatment (b).

Figure 4 shows TEM images of H_2_O_2_-treated ZnO NPs and XRD spectra before and after H_2_O_2_ treatment. As can be seen, the treatment with H_2_O_2_ created defects and grooves on the ZnO NP surface and the loosely bound, large aggregates. However, one should consider that the TEM images were taken after drying the samples and it is also possible that the aggregates could be the result of drying process on the TEM grid. The XRD spectra before and after H_2_O_2_ treatment are shown in Figure 4b. The broadened bands in the spectra after H_2_O_2_ treatment suggest that the grain size was reduced.

### Temperature-dependent size distribution of hydroxylated NPs

The TiO_2_ and ZnO NPs used in the study are poorly dispersed in dH_2_O. When suspensions of these NPs are prepared, large clusters and aggregates are observed. After 30 min of sonication, their dispersion is slightly improved in dH_2_O and after a short time (10 min) the NPs are precipitated.

**Figure 5:**
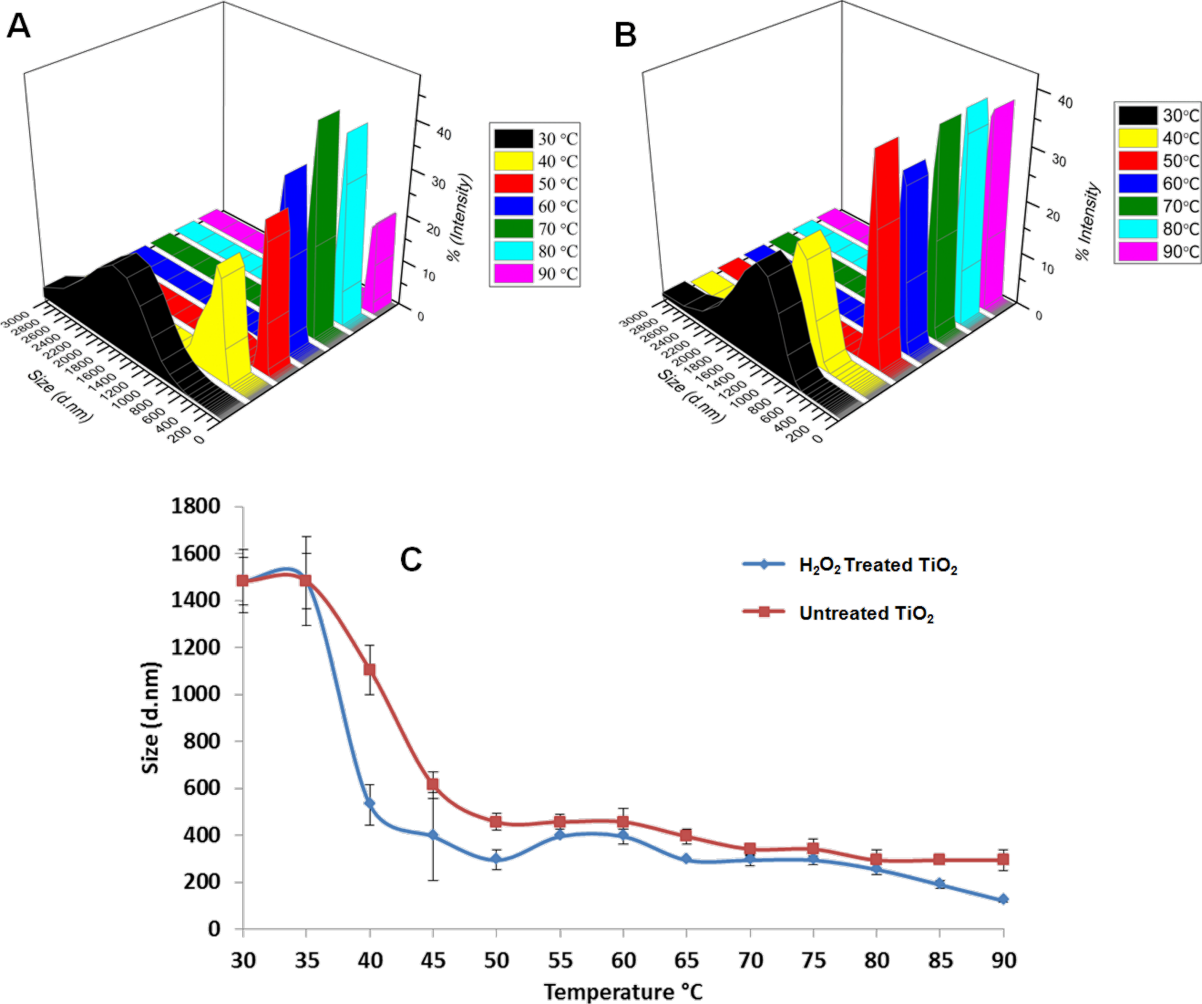
Temperature-dependent size change of H_2_O_2_-treated TiO_2_ NPs (a), untreated TiO_2_ NPs (b) and the size as a function of temperature change of untreated and H_2_O_2_ treated TiO_2_ NPs (c).

The effects of heating and treatment with hydrogen peroxide on the size distribution of the ZnO and TiO_2_ NPs were investigated by measuring the hydrodynamic radius of the NPs in their suspension in water. Figure 4 shows the temperature-dependent, hydrodynamic radius change of the untreated and hydroxylated TiO_2_ NPs. As mentioned above, the hydroxylation process removes possible impurities from the NP surface and increases the density of –OH groups on the surface. Therefore, the NPs are better dispersed in dH_2_O. Figure 5a shows the change in the hydrodynamic radius of the hydroxylated TiO_2_ NPs with increasing temperature. Between 30–40 °C, the TiO_2_ NP aggregates also exist in very large clusters (in the micrometer range) and with a large size distribution, and thus, the standard deviation of the measurement was also high. This is due to the presence of large clusters and hydroxylation provides slightly better dispersion in dH_2_O. As the temperature is further increased, the decrease in the hydrodynamic radius of the treated TiO_2_ NPs is clearly observed. At 90 °C, we obtained NPs of ≈120 nm, which we can consider as the primary NPs. It can be concluded that the most significant decrease in the hydrodynamic radius of the TiO_2_ NPs occurs after 80 °C. In order to confirm that these changes in the hydrodynamic radius of the TiO_2_ NPs are related to the hydroxylation or temperature effect, the experiment was repeated with the untreated TiO_2_ NPs. Figure 5b shows the temperature-dependent hydrodynamic radius change of the untreated TiO_2_ NPs. In this case, the hydrodynamic radius of the untreated TiO_2_ NPs decreases with increasing temperature, but the smallest size obtained was around ≈290 nm and the standard deviation of the measurement was higher, indicating a large size variation in the aggregates. The untreated TiO_2_ NP suspension contained large micrometer sized clusters in the range of 30–40 °C, and between 50–90 °C the size decreased to 300–500 nm.

In Figure 5c, the comparison of the change in the hydrodynamic radius of hydroxylated and untreated TiO_2_ NPs with increasing temperature is given. The hydrodynamic radius of the hydroxylated TiO_2_ NPs is smaller when compared to the untreated TiO_2_ NPs at each temperature interval. The large standard deviation in the case of the untreated TiO_2_ NPs indicates the high polydispersity of the particles in the suspension. The hydrodynamic radius of the hydroxylated TiO_2_ NPs decreased by 91.7% (from 1484 to 122 nm) and for the untreated TiO_2_ NPs by 80.1% (from 1484 to 295 nm) when the temperature was increased from 30 to 90 °C. After 60 °C, between 60–90 °C, this difference was observed more clearly. The hydrodynamic radius of the hydroxylated and the untreated TiO_2_ NPs was reduced to 69.1% and 35.5%, respectively.

The untreated ZnO NPs have lower dispersion in dH_2_O when compared to untreated TiO_2_ NPs. Even after 30 min of sonication, they are poorly dispersed and precipitated faster than TiO_2_ NPs. Thus, it becomes difficult to work with the untreated ZnO NPs in dH_2_O.

**Figure 6:**
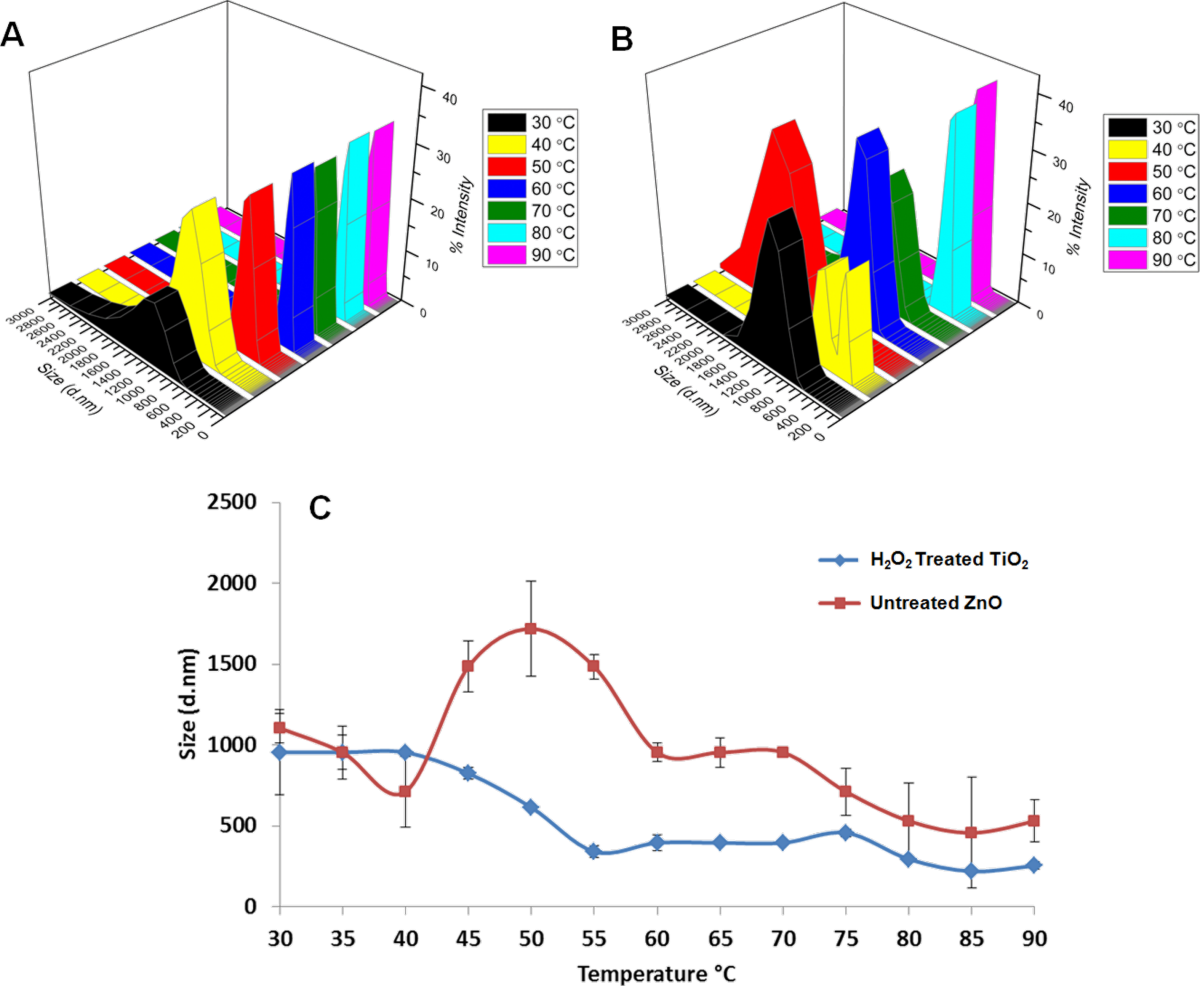
Temperature-dependent size change of H_2_O_2_-treated ZnO NPs (a), untreated ZnO NPs (b) and the change in size as a function of temperature for the untreated and H_2_O_2_-treated ZnO NPs (c).

The ZnO NPs were hydroxylated using the same procedure as was used for the TiO_2_ NPs and the effect of hydroxylation on the hydrodynamic radius of the NPs was much more apparent in the case of the ZnO NPs. By comparing with Figure 6a,b, it can be seen that the hydrodynamic radius of the hydroxylated ZnO NPs is significantly smaller than the untreated ZnO NPs at each temperature interval. The hydrodynamic radius of the ZnO NPs remains around 900–1200 nm until the temperature increases to 40 °C, but after that, the radius increases to ≈2000 nm at 50 °C and then decreases until a temperature of 90 °C is reached. This can be explained by the lack of dispersion of the untreated ZnO NPs in dH_2_O. The untreated ZnO NPs tend to form larger clusters than the treated TiO_2_ NPs in dH_2_O and precipitate much faster; therefore, the average size deviation is higher for the untreated ZnO NPs.

The hydroxylated ZnO NPs show a more gradual decrease in hydrodynamic radius with an increase in temperature. From Figure 6a, it can be seen that the hydroxylated ZnO NPs are around 1000 nm in the range of 30–40 °C and become smaller with increasing temperature, eventually reaching ≈280 nm at 90 °C. When the size of a single ZnO NPs (≈98 nm) is considered, the obtained ≈280 nm of hydrodynamic radius indicates that the NPs form aggregates consisting of a few NPs. It is more clear from Figure 6c that when the hydroxylation and heating processes are combined, the hydrodynamic radius of the ZnO NPs is reduced. At 90 °C, most of the aggregates in the ZnO suspension are broken down and the primary particles are obtained. Figure 6c shows the change in the size of untreated and hydroxylated ZnO NPs at each temperature interval. Due to the very low dispersion of untreated ZnO NPs in dH_2_O, the size variation is very high. The uncontrolled precipitation of untreated ZnO NPs leads to the irregular size change with increasing temperature. However, in the case of the hydroxylated ZnO NPs, there is a steady decrease in the size of the NPs with increasing temperature. When the temperature is increased from 30 to 90 °C, the decrease in the hydrodynamic radius of the hydroxylated ZnO NPs was 73.2% and 51.9% for the untreated ZnO NPs. After 60 °C, the profile change in the size of both hydroxylated and untreated ZnO NPs was observed to be similar. From 60 to 90 °C, the decrease in the size of the hydroxylated ZnO NPs (from 955 nm to 255 nm) is 35.6% and for the untreated ZnO NPs, decreases (from 1106 nm to 531 nm) by 44.3%. The percent decrease in the case of the untreated ZnO NPs is higher but the final size of the untreated ZnO (531 nm) is more than 50% larger than the size of hydrogen peroxide-treated ZnO NPs (255 nm).

## Conclusion

In this study, a new treatment process to overcome the problem of agglomeration of ZnO and TiO_2_ NPs by treating them with hydrogen peroxide and heating up to 90 °C was demonstrated. The ZnO and TiO_2_ NPs were successfully dispersed in aqueous media without using a surfactant or other dispersing agents. Since H_2_O_2_ is decomposed into H_2_O and O_2_ during the NP treatment, the process is environmentally friendly. The hydrogen peroxide treatment washes the surface of NPs and helps to generate additional –OH groups on the NP surface. The same procedure applied to the untreated NPs in dH_2_O caused a very small change in their dispersion. When the hydroxylation process and the temperature treatment between 30–90 °C were combined, it was observed that the hydrodynamic radius of the NPs decreased from a few micrometers (1–2 μm) to a few hundred namometers (200–300 nm). For both ZnO and TiO_2_ NPs, the hydrodynamic radius decreased with the influence of both hydroxylation and heating. The hydrodynamic radius of the hydroxylated TiO_2_ NPs dramatically decreased to ≈120 nm after 80 °C. The hydrodynamic radius of the untreated and hydroxylated ZnO NPs showed different trends with increasing temperature. Due to the poor dispersion and the formation of very large aggregates, the size of the untreated ZnO NPs did not decrease systematically with the increase in temperature. Heating alone was not enough to break down the aggregates of the untreated ZnO NPs; however, when heating was combined with the hydroxylation process, the hydrodynamic radius of the ZnO NPs was decreased to ≈280 nm after 75 °C. The presented approach may allow for the dispersion of MONPs in water without contamination.
